# Characterization and Protective Activity of Monoclonal Antibodies Directed against *Streptococcus suis* Serotype 2 Capsular Polysaccharide Obtained Using a Glycoconjugate

**DOI:** 10.3390/pathogens8030139

**Published:** 2019-09-07

**Authors:** Guillaume Goyette-Desjardins, Sonia Lacouture, Jean-Philippe Auger, René Roy, Marcelo Gottschalk, Mariela Segura

**Affiliations:** 1 Research Group on Infectious Diseases in Production Animals, Faculty of Veterinary Medicine, University of Montreal, St-Hyacinthe, QC J2S 2M2, Canada; guillaume.goyette-desjardins@umontreal.ca (G.G.-D.); sonia.lacouture@umontreal.ca (S.L.); jean-philippe.auger.1@umontreal.ca (J.-P.A.); marcelo.gottschalk@umontreal.ca (M.G.); 2 Swine and Poultry Infectious Diseases Research Centre, Saint-Hyacinthe, QC J2S 2M2, Canada; 3 Canadian Glycomics Network (GlycoNet), University of Alberta, Edmonton, AB T6G 2G2, Canada; roy.rene@uqam.ca; 4 Department of Chemistry, Université du Québec à Montréal, Montreal, QC H3C 3P8, Canada

**Keywords:** *Streptococcus suis*, serotype 2, capsular polysaccharide, monoclonal antibody, opsonophagocytosis

## Abstract

*Streptococcus suis* serotype 2 is an encapsulated bacterium and an important swine pathogen. Opsonizing antibody responses targeting capsular polysaccharides (CPSs) are protective against extracellular pathogens. To elucidate the protective activity of monoclonal antibodies (mAbs) directed against *S. suis* serotype 2 CPS, mice were immunized with a serotype 2 CPS-glycoconjugate and three hybridomas were isolated; of which, two were murine IgMs and the other a murine IgG1. Whereas the IgMs (mAbs 9E7 and 13C8) showed different reactivity levels with *S. suis* serotypes 1, 1/2, 2 and 14, the IgG1 (mAb 16H11) was shown to be serotype 2-specific. All mAbs targeted the sialylated chain of the CPSs. Using an opsonophagocytosis assay, the IgMs were opsonizing towards the *S. suis* serotypes to which they cross-react, while the IgG1 failed to induce bacterial elimination. In a model of mouse passive immunization followed by a lethal challenge with *S. suis* serotype 2, the IgG1 and IgM cross-reacting only with serotype 14 (mAb 13C8) failed to protect, while the IgM cross-reacting with serotypes 1, 1/2, and 14 (mAb 9E7) was shown to be protective by limiting bacteremia. These new mAbs show promise as new *S. suis* diagnostic tools, as well as potential for therapeutic applications.

## 1. Introduction

*Streptococcus suis* is an encapsulated Gram-positive bacterium and one of the most important bacterial pathogens in the porcine industry, resulting in important economic losses [1]. To date, the capsular polysaccharide (CPS) antigenic diversity has allowed the classification of *S. suis* in 35 serotypes. *S. suis* serotype 2 is considered the most virulent, being the serotype most frequently isolated from clinical samples and associated with disease in swine in most countries [2]. *S. suis*, mainly serotype 2, is also an important emerging zoonotic agent for people in close contact with pigs or pig-derived products [2]. Of the various manifestations of the disease, septicemia and meningitis are by far the most remarkable, but other pathologies have also been observed [1]. Research has been ongoing for decades in the hope of developing an efficient commercial vaccine to protect post-weaning pigs against *S. suis* infections. Yet, to our knowledge, no such vaccine with proven efficacy is available [3].

It is well known that the thick-surface associated CPS confers protection to *S. suis* against the immune system, notably by resisting phagocytosis [4,5]. Thus, as with other encapsulated pathogens such as *Streptococcus pneumoniae*, *Neisseria meningitidis*, *Haemophilus influenzae*, and Group B *Streptococcus*, antibodies directed against the CPS are highly opsonizing and protective [6,7]. However, due to their carbohydrate nature, CPSs are generally considered poorly immunogenic since they are unable to recruit T cell help for B cell functions [8]. A well known strategy to overcome the T-independent (TI) nature of CPSs is to conjugate the polysaccharide onto a carrier protein that will provide T-dependent (TD) epitopes to allow a potent vaccine response against the polysaccharide [9,10]. Thus, we recently created a first glycoconjugate vaccine, made from *S. suis* serotype 2 CPS coupled to tetanus toxoid (TT) by reductive amination, and found it to induce opsonizing anti-CPS antibodies in mice and to be protective in pigs against a challenge carried out with this serotype [11]. 

**Figure 1:**
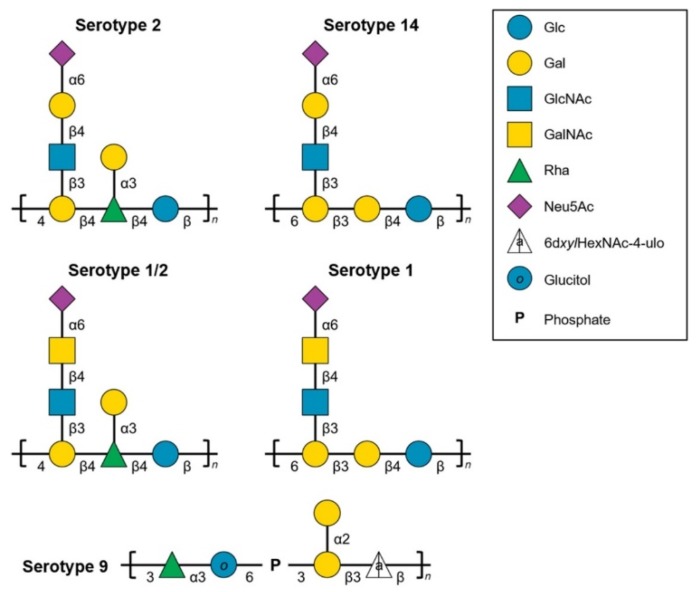
Comparison of reported structures for the capsular polysaccharide repeating units of *S. suis* serotypes 2 [12], 1 [14], 1/2 [14], 14 [13], and 9 [15]. Monosaccharide symbols follow the SNFG (Symbol Nomenclature for Glycans) system [21]. Abbreviations: D-glucose (Glc), D-galactose (Gal), *N*-acetyl-d-glucosamine (GlcNAc), *N*-acetyl-d-galactosamine (GalNAc), L-rhamnose (Rha), *N*-acetyl-d-neuraminic acid (Neu5Ac), 2-acetamido-2,6-dideoxy-β-d-*xylo*-hexopyranos-4-ulose (6d*xyl*HexNAc-4-ulo), and phosphate (P).

Currently, exact structures for the repeating units (RUs) of the CPS of nine different serotypes have been reported, including those for serotypes 2, 14, 1, 1/2, 9, 3, 18, 7, and 8 of *S. suis* [12,13,14,15,16,17]. Serotypes 2, 14, 1, and 1/2 RUs are formed of acidic branched hexa- or heptasaccharides and all possess α2,6-linked sialic acid (Neu5Ac) at their non-reducing ends (Figure 1). Serotype 9 RU is non-sialylated and formed of an acidic branched tetrasaccharide (Figure 1). Serotypes 2 and 1/2 and serotypes 1 and 14 share common epitopes and present cross-reactions when serotyping by the co-agglutination method [2]. Alternatively, serotyping by PCR cannot resolve those cross-reactions either, as these serotypes do not possess unique *cps* genes [2,18]. Indeed, serotypes 2 and 14 both possess a β-galactose (Gal) in their side chain that is found *N*-acetylated (GalNAc) in serotypes 1 and 1/2 [14]. More recently, our group has shown that a single amino acid polymorphism in the glycosyltransferase CpsK defines the enzyme predilection for Gal or GalNAc and therefore determines CPS composition and strain serotype [19,20]. Still, very little is known about *S. suis* type 2 CPS protective epitopes. A previous study aimed at explaining the serological characteristics of *S. suis* serotypes 2, 1, 1/2, and 14 using purified CPSs and rabbit type-specific sera showed that the sialic acid-bearing side chain and, most importantly, that its terminal sialic acid, constitutes a major immunogenic structure for serotype 2 CPS [14].

Monoclonal antibodies (mAbs) are nowadays common useful tools employed in many settings, ranging from the study of bacterial virulence factors to therapeutic applications. Previously, Charland et al. reported the characterization and protective activity of mAb Z3 which was found to be directed to the CPS of *S. suis* serotype 2; interestingly, it also reacted with the CPS of serotypes 1 and 1/2 [22]. In that study, although more than 3000 clones were tested following hyperimmunization of mice with formaldehyde-inactivated bacteria, only the mAb Z3 was found to react with the CPS, which suggests a very low frequency of CPS-specific clones. The mAb Z3 was also shown to present a specificity for the terminal sialic acid [22]. It has also been well demonstrated that *S. suis* serotype 2 CPS is non-immunogenic, even when expressed at the bacterial surface during an infection or in the presence of strong adjuvants such as water-in-oil emulsions like TiterMax Gold^®^ and STIMUNE^®^ [11,23,24,25].

Our hypothesis was that a glycoconjugate (made from *S. suis* serotype 2 CPS coupled to TT) improves frequency and diversity of serotype 2 CPS-specific B cell clones and thus hybridomas after fusion with a myeloma cell line. Therefore, the aim of this study was to obtain, characterize, and study the protective activity of murine mAbs targeting *S. suis* serotype 2 CPS. In turn, these new mAbs were also used to help define the protective epitopes of *S. suis* serotype 2 CPS.

## 2. Results

### 2.1. Glycoconjugate Preparation

The preparation of glycoconjugate employed in this study, consisting of *S. suis* serotype 2 CPS coupled to tetanus toxoid by reductive amination with a molar ratio of 2 CPS: 1 TT, and its complete characterization (coupling efficiency, *in vivo* antibody response, opsonization, and protection) has been previously reported in [11].

### 2.2. Characterization of mAbs

The screening steps against *S. suis* type 2 CPS of mAb production by ELISA led to the selection of 3 positive clones: 9E7 (IgM), 13C8 (IgM), and 16H11 (IgG1) out of 700 clones tested. In addition, previously described mAb Z3 was included in this study as a protective positive control and for dot-blot experiments regarding CPS specificity [22]. Although it had originally been reported to be an IgG2b, mAb Z3 was found later to be of the IgM isotype (Table S1); this discrepancy could be explained by the low specificity of the commercial reagents that were available at the time of the study by Charland et al. [22]. 

**Figure 2:**
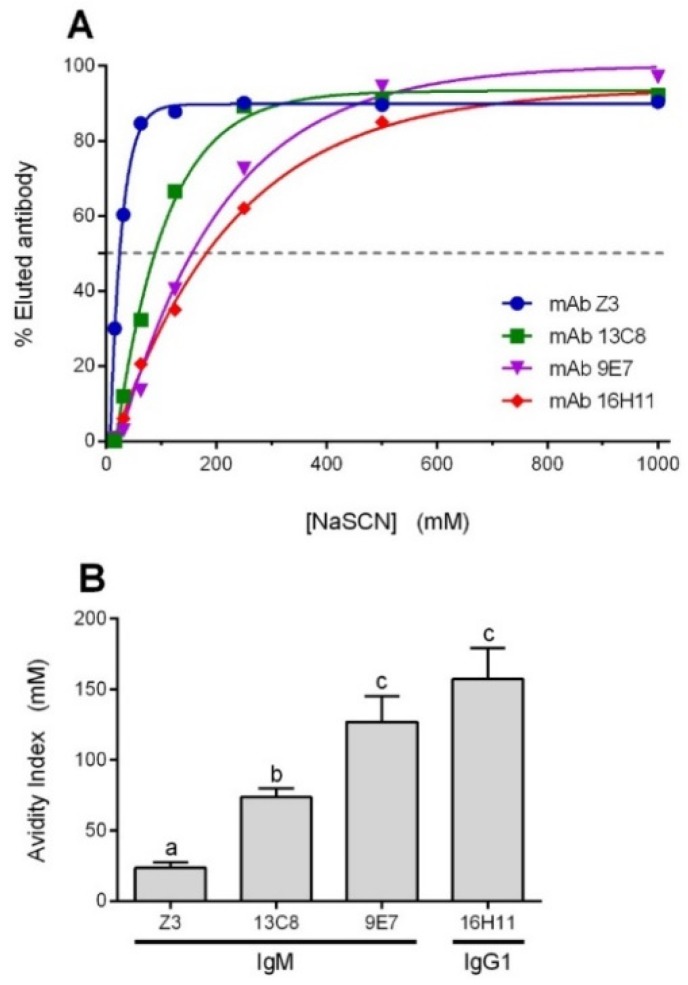
Avidity of mouse mAbs toward *S. suis* serotype 2 CPS. (**A**) Representative NaSCN elution curves of type 2 CPS-specific mAbs. (**B**) Avidities were expressed as avidity index (i.e., the millimolar concentration of NaSCN eluting 50% of type 2 CPS-specific mAbs). Dilutions of mAbs were chosen to produce absorbance readings at the top of the titration curve (A450 ≥ 1.0). Data are expressed as mean ± SEM of at least 3 repetitions. Significant difference between mAbs were determined by ANOVA followed by the Tukey test and is indicated by different letters (*p* < 0.05); accordingly, identical letters between groups indicate no difference.

In order to evaluate the avidity of our mAbs toward type 2 CPS, a thiocyanate elution assay was performed using an anti-CPS indirect ELISA. As shown in Figure 2, the mAb with the strongest antibody avidity index (AI) was found to be the IgG1 16H11, followed closely by the IgM 9E7, while IgMs 13C8 and Z3 showed intermediate and low avidities, respectively.

### 2.3. Characterization of the S. suis Capsular Epitopes Recognized by the mAbs

**Figure 3:**
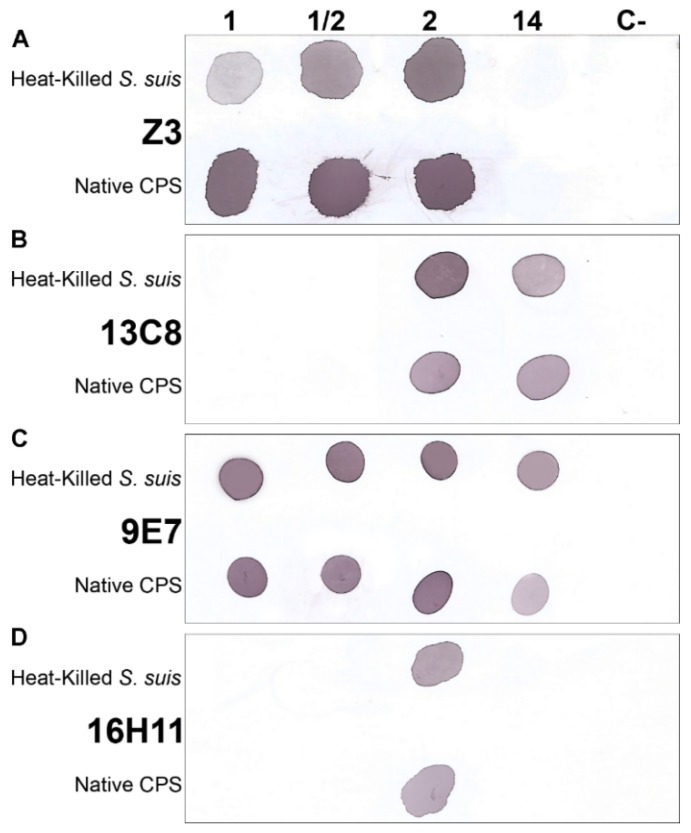
Serotype specificity of mAbs recognizing *S. suis* CPS. Dot-blot analyses with 1 × 10^7^ CFU of heat-killed bacteria or 10 µg of native CPS from serotypes 1, 1/2, 2, 14 or 9 (negative control) using different mouse mAb culture supernatants whose titers ranged from 48 to 88, with an average (±SD) titer of 65.5 ± 16.9, as determined by anti-CPS indirect ELISA: mAb Z3 (**A**), mAb 13C8 (**B**), mAb 9E7 (**C**), or mAb 16H11 (**D**).

Since mAb Z3 was originally reported to recognize the sialylated CPSs of *S. suis* serotypes 1, 1/2 and 2 [22], we decided to conduct dot-blot analyses using both heat-killed bacteria and purified CPSs of those serotypes but also serotype 14 of *S. suis*, since their CPS RU structures are highly similar (Figure 1). As aforementioned, they all possess sialic acid and are known to present cross-reactions when serotyping is performed with rabbit antisera [14]. Serotype 9 was included as a negative control since its CPS RU structure is unrelated to those of the other studied serotypes (Figure 1). Results of the dot-blots analyses are shown in Figure 3. 

As reported [22], IgM mAb Z3 strongly recognized the CPSs of serotypes 1, 1/2, and 2, but not that of serotype 14 (Figure 3A). In contrast, IgM mAb 13C8 gave strong reactions with serotypes 2 and 14, but did not recognize the CPSs of serotypes 1 and 1/2 (Figure 3B). The IgM mAb 9E7 recognized all four CPSs (1, 1/2, 2, and 14) (Figure 3C). Interestingly, IgG1 mAb 16H11 reacts solely with serotype 2 (Figure 3D).

The cross-reactions between *S. suis* serotypes 2 and 14 (IgM mAbs 13C8 and 9E7) were unexpected based on published serological tests [23,26], especially in the absence of reactions with serotypes 1 and 1/2 as is the case for mAb 13C8. Using serotypes 2 and 14 whole bacterial cell extracts in Western blots, no positive signal was observed, which means that mAbs 13C8 and 9E7 do not react with protein or cell-wall antigens (data not shown). ELISA screening and dot-blot analyses showed for all mAbs a strong specificity toward sialylated CPSs but not against their desialylated counterparts (Figure S1), as previously reported for IgM mAb Z3 [22]. Finally, none of the mAbs recognized the heat-killed bacteria or the CPS of serotype 9, used as control (Figure 3).

### 2.4. Functional Activity of mAbs

**Figure 4:**
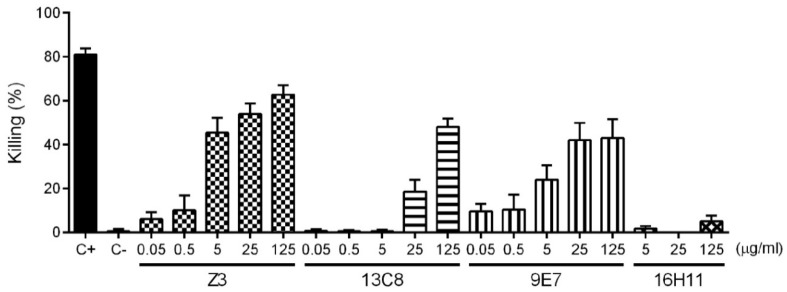
Opsonophagocytic killing of *S. suis* serotype 2 strain S735 by different concentrations of purified mouse mAbs. Different concentrations (indicated on the *x*-axis in µg/mL) of purified mAb diluted in complete culture medium and a bacterial MOI of 0.1 were added to whole blood from naive CD-1 mice to perform the assay. Viable bacterial counts were performed after 2 h of incubation. To determine bacterial killing, viable bacterial counts from tubes incubated with mAbs were compared to those incubated without antibodies (complete culture medium + blood + bacteria). An IgM isotype control (C−) was used as a negative control for the mAbs. Serum from an hyperimmune rabbit was used as a positive control (C+) and compared to control naive rabbit serum to determine bacterial killing. Results are expressed as percent of bacterial killing for each dose, represented as mean ± SEM of at least 3 repetitions.

Opsonophagocytosis is thought to be the main mechanism responsible for clearing encapsulated Gram-positive bacteria via both Fc-receptor-mediated and/or C1q- and/or C3b-mediated (complement-dependent) phagocytosis [27,28,29,30]. Therefore, the functional activity of the mAbs against *S. suis* serotype 2 was investigated by performing a standardized opsonophagocytosis assay (OPA) test using whole blood from mice as effector cells [11,31]. Firstly, a dose-response experiment using purified mAbs Z3, 13C8, 9E7, and 16H11 was performed (Figure 4). All IgMs (namely mAbs Z3, 13C8 and 9E7) were found to be opsonizing, with a maximum killing value of approximately 50–60% at the highest concentration used. These data indicate that these purified mAbs are functional and able to mediate the clearance of *S. suis* serotype 2 by opsonophagocytosis *in vitro*. Since the opsonizing capacity of IgM mAb 13C8 dropped faster than that of IgM mAbs Z3 and 9E7, opsonic indexes (OIs), corresponding to the dose of mAb (in µg/mL) yielding 50% of maximal killing value, were calculated. The mAb 13C8 has an OI of 27.8 µg/mL, an opsonizing capacity approximately 6 to 12 times lower than that of mAb 9E7 (OI = 4.35 µg/mL) or mAb Z3 (OI = 2.35 µg/mL), respectively. In contrast to IgMs, murine IgG1 mAb 16H11 was found to be not opsonizing, with killing values below 5%, even with a higher dose of 500 µg/mL (Figure 4 and data not shown).

**Figure 5:**
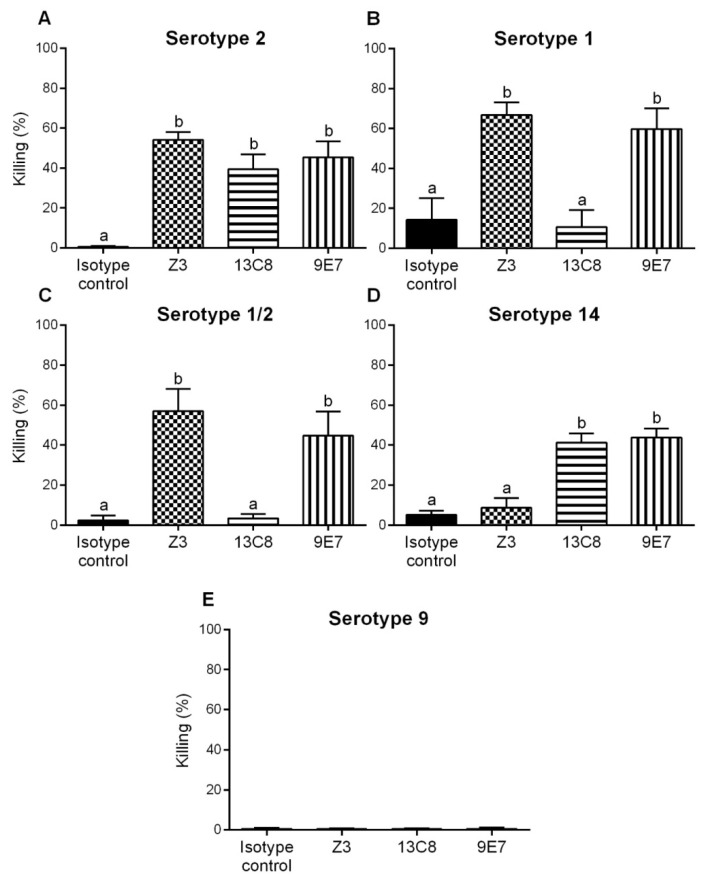
Opsonophagocytic killing of different *S. suis* serotypes by purified mouse mAbs. Purified mAbs (125 µg/mL) diluted in complete culture medium and a bacterial MOI of 0.1 were added to whole blood from naive CD-1 mice to perform the assay against *S. suis* serotype 2 strain S735 (**A**), serotype 1 strain 1178027 (**B**), serotype 1/2 strain 2651 (**C**), serotype 14 strain DAN13730 (**D**), and serotype 9 strain 1273590 (**E**), used as a negative control. Viable bacterial counts were performed after 2 h of incubation. To determine bacterial killing, viable bacterial counts from tubes incubated with mAbs were compared to those incubated without antibodies (complete culture medium + blood + bacteria). An IgM isotype control was used as a negative control for the mAbs. Results are expressed as percent of bacterial killing for each dose, represented as mean ± SEM of at least 3 repetitions. Significant difference between mAbs and/or isotype control were determined by ANOVA followed by the Tukey test and is indicated by different letters (*p* < 0.05).

Since the three IgMs were opsonizing with *S. suis* serotype 2, we wanted to validate whether the cross-reactions observed by dot-blot with the CPSs of other serotypes (Figure 3) were specific and of clinical importance. To this end, OPAs were repeated using a single dose of 125 µg/mL of mAb against strains of serotypes 1, 1/2, and 14. A serotype 9 strain and an IgM isotype control were included as negative controls. As shown in Figure 5, OPA results correlated with cross-reactions observed in dot-blot analyses. The IgM mAb Z3 induced opsonophagocytosis of *S. suis* types 1, 1/2, and 2 but not of type 14. The IgM mAb 13C8 opsonized types 2 and 14 only; while IgM mAb 9E7 opsonized all 4 serotypes. The absence of significant killing (*p* > 0.05) by either the isotype control (Figure 4 and Figure 5) or by mAb-opsonization of *S. suis* serotype 9 (Figure 5E) demonstrated the specificity of these mAbs toward the sialylated CPSs of *S. suis* serotypes 2, 1, 1/2, and/or 14. 

**Figure 6:**
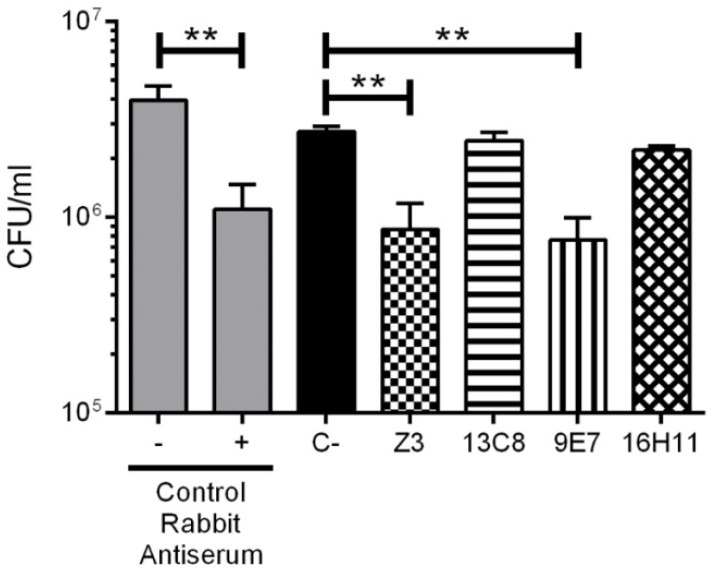
MAb-induced agglutination of *S. suis* serotype 2 strain S735. Purified mAbs (125 µg/mL) diluted in complete culture medium were added to 2.5 × 10^5^ CFU/mL. Viable bacterial counts were performed after 2 h of incubation and represented as mean ± SEM of at least 3 repetitions. Significant differences between mAbs and/or isotype control (C−) were determined by ANOVA followed by the Tukey test are denoted as follows: ** *p* < 0.01.

Additionally, the ability of these mAbs to induce bacterial agglutination was also investigated, since this confounding mechanism could also lead to a positive result in the OPA assay (Figure 6). The agglutination assay was performed in the same fashion as the OPA assay, minus the whole blood (source of phagocytic cells). Both highly opsonic IgM mAbs Z3 and 9E7 produced a significant reduction in the viable counts of the *S. suis* serotype 2 strain (*p* < 0.001), which suggests bacterial agglutination. To the contrary, no reduction in viable counts (*p* > 0.05) was observed with the opsonic IgM mAb 13C8 nor with the non-opsonic IgG1 mAb 16H11, indicating that these two mAbs do not agglutinate *S. suis* serotype 2.

**Figure 7:**
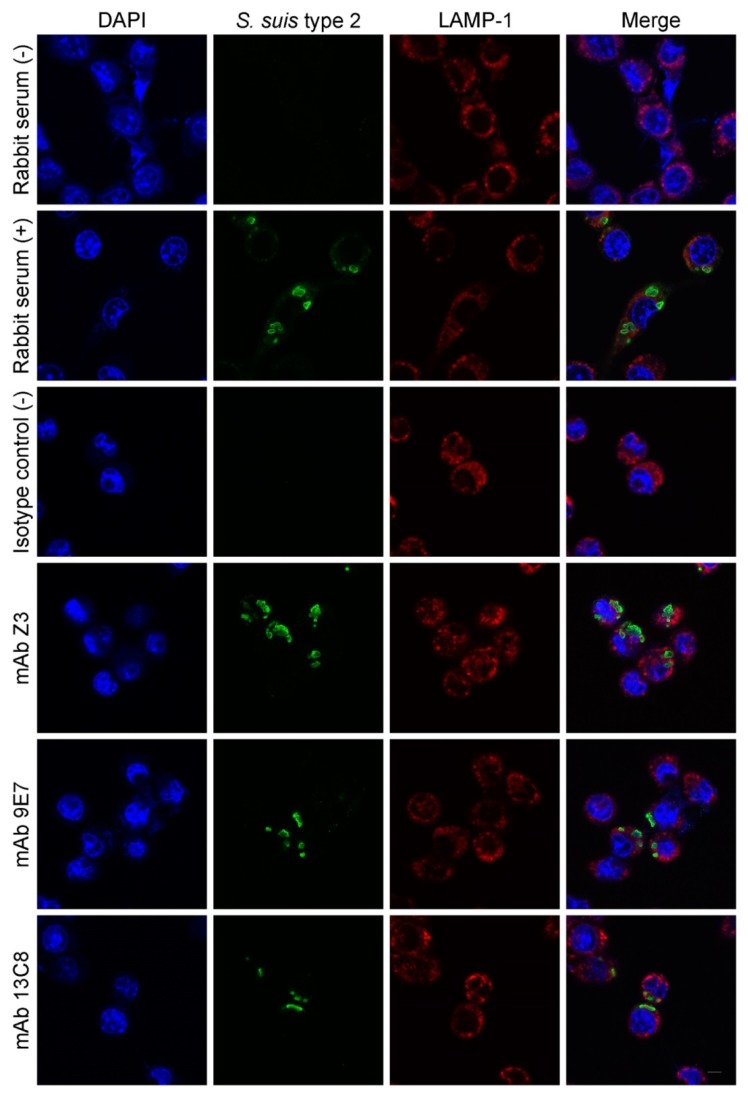
Phagocytosis of mAb-opsonized *S. suis* serotype 2 by confocal laser scanning microscopy. After a bacteria–cell contact of 1 h, cells were fixed and labelled with serum against *S. suis* (Alexa-Fluor 488, green) and a mAb specific for LAMP-1 (Alexa-Fluor 647, red). DAPI was used to stain nuclei or bacterial DNA (blue). Merge of optical sections shows localization of opsonized *S. suis* inside LAMP-1+ vacuoles. An IgM isotype control was used as a negative control. *S. suis* opsonized with rabbit sera (either + or −) were also included as controls. Staining controls were performed and shown in Figure S3. Scale bar = 5 μm.

Using J774 murine macrophages and pre-opsonized *S. suis* serotype 2, immunofluorescence studies by confocal microscopy were performed to investigate whether or not the opsonic IgM mAbs can induce immunoclearance of *S. suis* through opsonophagocytosis (Figure 7). Bacteria were pre-opsonized in the presence of 125 µg/mL of purified mAb and 40% (*v*/*v*) fresh serum from naïve mice as a source of complement. When *S. suis* serotype 2 was pre-opsonized by an IgM isotype control, no bacteria could be found inside macrophages (Figure 7), illustrating well the extent of encapsulated *S. suis*-resistance to phagocytosis [4,5].

Internalized bacteria are directed into phagosomes that might fuse with lysosomes in order to mature into phagolysosomes. Throughout this maturation process, phagosomes will acquire membrane markers from early and late endosomes, one of which being LAMP-1 [32]. When IgM mAbs Z3 and 9E7 were employed to pre-opsonize *S. suis* serotype 2, large bacterial clumps could be found within phagolysosomal (LAMP-1^+^) vacuoles (Figure 7), which was also the case with serotype-specific rabbit antiserum used as positive control. When IgM mAb 13C8 was used for pre-opsonisation, individual bacteria and chains can be observed inside the phagolysosomal vacuoles of macrophages (Figure 7). Overall, these results indicate that our IgM mAbs do induce opsonophagocytosis *in vitro*, since both bacterial coating and bacterial agglutination by IgM can trigger capture by phagocytes [27,33].

### 2.5. Protective Potential of mAbs

**Figure 8:**
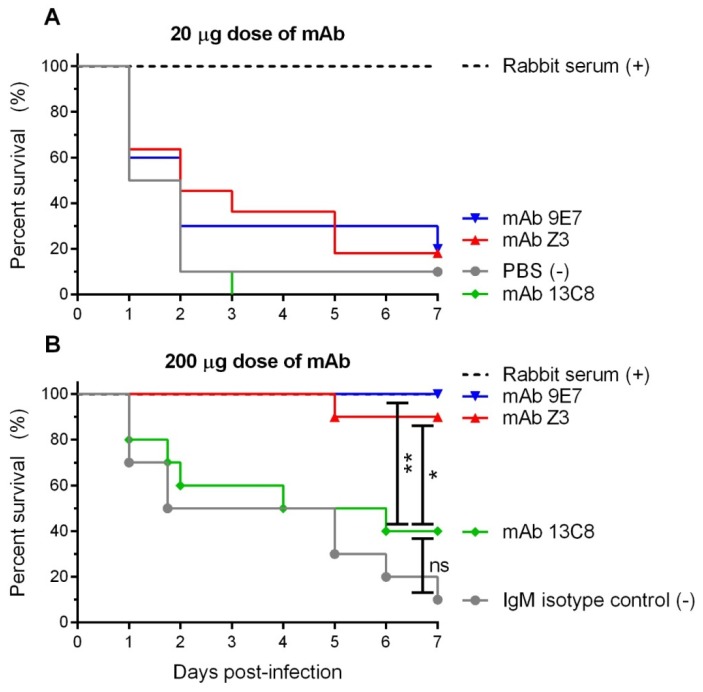
Passive immunization of CD-1 mice with mAbs and survival after lethal challenge with *S. suis* type 2. Mice (n = 10) received doses of 20 µg ((**A**), pre-trial) or 200 µg (**B**) of purified mAbs intraperitoneally 1 h prior to the challenge. Mice were then challenged with a LD90 dose of 1 × 10^8^ CFU of *S. suis* serotype 2 strain P1/7 by intraperitoneal inoculation. Negative controls used for protection were PBS (**A**) or an IgM isotype control (**B**). Type 2 specific rabbit antiserum was used as the positive control. Significant differences between groups were determined using the log-rank (Mantel-Cox) test and are denoted as follows: Not significant (ns), * *p* < 0.05; ** *p* < 0.01.

Since the IgM mAbs 9E7 and 13C8 were able to mediate the clearance of *S. suis* serotype 2 by opsonophagocytosis *in vitro*, their protective capacity *in vivo* was evaluated by passive immunization. The IgM mAb Z3 has previously been shown to be protective when used for pre-opsonizing bacteria prior to intraperitoneal infection in mice [22]. In this study, to better evaluate the *in vivo* activity of the IgM mAbs, we developed a passive immunization assay in CD-1 mice using intraperitoneal administration of the antibody 1 h prior to a lethal challenge with a LD90 dose of 1 × 10^8^ CFU of virulent *S. suis* serotype 2 strain P1/7 intraperitoneally (Figure 8). In a first pre-trial protection assay, a dose of 20 µg was chosen based on other reports investigating mAbs against *S. pneumoniae* CPSs [34,35,36]. Two days post-infection (p.i.), 9 out of 10 mice in the negative control group presented serious clinical signs of *S. suis* disease and had to be euthanized (Figure 8A). All mice injected with the polyclonal rabbit serum (used as positive control) survived and did not show any clinical signs. However, no significant protection (*p* > 0.05) was observed in mice passively immunized with either IgM mAbs 9E7, 13C8 or Z3 (the latter included as control) when administrated at a dose of 20 µg (Figure 8A).

**Figure 9:**
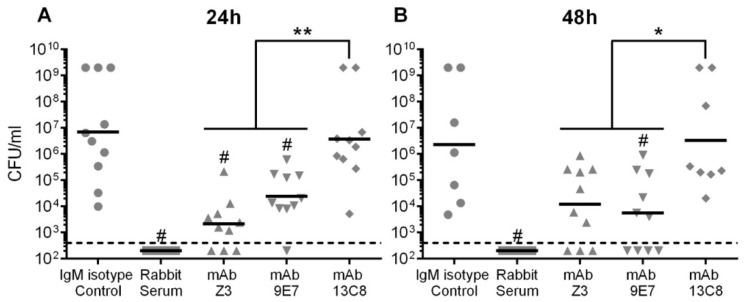
Passive immunization of CD-1 mice with 200 µg of mAbs and blood bacterial burden after lethal challenge. Mice (n = 10) received a dose of 200 µg of purified mAbs intraperitoneally 1 h prior to the challenge with 1 × 10^8^ CFU of *S. suis* serotype 2 strain P1/7 by intraperitoneal inoculation. Type 2 specific rabbit antiserum was used as the positive control. Bacterial counts were determined in serial dilutions of 5 µL of blood obtained from the tail vein at 24 h (**A**), 48 h (**B**), post-infection. Data of individual mice are presented as CFU/mL with the median. Significant differences between groups were determined using the Kruskall–Wallis ANOVA on ranks followed by the Dunn procedure and are denoted as follows: * *p* < 0.05; ** *p* < 0.01. # indicates bacteremia levels significantly different from those of the isotype control group (*p* < 0.05).

Therefore, we decided to increase the mAb dose to ensure that a failure in protection was not due to a low antibody concentration. In the second protection assay, mice received a dose of 200 µg of IgM mAb. Seven days p.i., all mice from the positive control (rabbit antisera) survived, whereas the IgM isotype control group presented 90% of mortality (Figure 8B). Mice injected with IgM mAbs Z3 or 9E7 showed 90% and 100% of protection 7 days p.i., respectively. On the other hand, mice treated with IgM mAb 13C8 showed 40% of protection which was not significantly different than the isotype control (*P* = 0.23). Indeed, at 7 days p.i., protection conferred by IgM mAbs Z3 and 9E7 was found to be significantly higher (*p* < 0.05) than that of IgM mAb 13C8. In agreement with protection levels, at 24 h p.i. mice in the isotype control group showed strong levels of bacteremia that were also sustained 48 h p.i. (Figure 9). Mice treated with the rabbit antiserum were able to clear the bacteria from circulation as soon as 24 h p.i. (Figure 9). At 24 h p.i., mice treated with IgM mAbs Z3 or 9E7 showed significantly lower bacteremia than the isotype control group (*p* ≤ 0.005) (Figure 9A). At 48 h p.i., mice treated with IgM mAbs Z3 or 9E7 also presented a considerable reduction in bacteremia compared to the negative control group, although the difference was significant only for IgM mAb 9E7 (*p* = 0.027) (Figure 9B). In contrast to these two IgMs, bacteremia levels in mice treated with IgM mAb 13C8 were comparable to those of the control group at both time points (Figure 9). 

## 3. Discussion

By employing a glycoconjugate made from *S. suis* serotype 2 CPS covalently linked to TT [11], we describe for the first time a murine IgG1 mAb directed to the CPS of *S. suis* serotype 2. Interestingly, this mAb was solely specific for serotype 2 CPS. Due to the cross-reactions between serotype 2 and serotype 1/2, two antisera (anti-serotype 1 and anti-serotype 2) must be employed to properly identify these serotypes. Therefore, the discovery of a mAb strictly specific for the serotype 2 of *S. suis* could result now in a one-step identification of these strains for diagnostic purposes.

In addition, this strategy also allowed the obtaining of two additional new murine IgMs, each recognizing distinct epitopes of the *S. suis* serotype 2 CPS and cross-reactive epitopes of serotypes 1, 1/2, and/or 14. All reactions were found to be specific towards the sialic acid and/or sialic acid-bearing side chain. Finally, while the IgG1 (mAb 16H11) was unable to promote bacterial immunoclearance *in vitro* or *in vivo* (data not shown), the IgMs were opsonizing and/or agglutinating *in vitro* and/or protective *in vivo*, yet at different degrees (Table 1). While it was previously found that a glycoconjugate vaccine made from *S. suis* serotype 2 CPS induces opsonizing anti-CPS antibodies in mice and protected pigs against this serotype [11], in this study it was demonstrated by employing mAbs that the *in vivo* protection is indeed due to the opsonizing capacities of those anti-CPS antibodies. In addition, IgM mAbs that were agglutinating (Z3 and 9E7) were positively associated with protection *in vivo*, as agglutination contributes greatly to complement activation (see below). Yet, mAb 13C8, which was non-agglutinating and not significantly protective, still was able to trigger phagocytosis of *S. suis* serotype 2 by macrophages and to trigger its killing in our OPA assay. We have thus been able to show that IgM mAbs are opsonizing and lead to bacterial clearance through opsonophagocytosis. In turn, this reflects the great potential of polysaccharides as vaccine antigens in preventing infections by extracellular bacteria.

It is now well known that *S. suis* serotype 2 CPS is particularly non-immunogenic, even when associated at the bacterial surface in the course of a primary or booster experimental infection [11,23,24,25,37,38]. This low immunogenicity results in low IgM titers and undetectable IgG titers. While one likely explanation is that CPSs are considered as TI antigens, purified CPSs from other streptococci are known to induce not only IgM but also IgG protective antibody responses [25,39,40]. The low immunogenicity of *S. suis* serotype 2 CPS cannot be explained by the presence of sialic acid, known to possess immunomodulatory properties [25]. Nonetheless, as with other encapsulated bacteria, conjugation of CPS to a carrier protein might help the induction of a stronger antibody response with TD features such as antibody class switching, affinity/specificity maturation, and immunological memory [8,9]. Here, the use of a glycoconjugate yielded two IgMs and one IgG against *S. suis* serotype 2 CPS, which is an improvement compared to the previous report by Charland et al. where whole bacteria was used as immunogen and only one IgM was obtained [22]. 

The obtained IgG mAb belongs to the IgG1 subclass, known to be privileged during a Th2 immune response. This isotype switch could be favored by the immunization strategy, the mouse genetic background, the immunological properties of the TT protein itself and/or the adjuvant used. For instance, TiterMax Gold^®^ is a squalene-based water-in-oil emulsion adjuvant which is known to yield either mixed Th1/Th2 or Th2-polarized humoral responses in mice [11,41,42]. The protein TT has been reported to induce a cytokine profile compatible with a type 2 response [43]. Other immunization strategies, such as employing either a different mouse strain, adjuvant or carrier protein for the glycoconjugate, could be used to obtain other IgG subclasses. As TD antibody responses are known to involve processes such as affinity maturation, this could explain why the IgG1-mAb 16H11 possesses such unique specificity to only *S. suis* serotype 2 CPS and a high avidity. Indeed, murine IgG1 has been previously reported to possess the highest mean antigen-binding affinity of all subclasses [44,45]. Nevertheless, due to its IgG1 nature, this mAb was unable to induce *S. suis* elimination by opsonophagocytosis *in vitro* and thus protection *in vivo* (Figure S2). Functional studies using mouse mAbs *in vitro* have shown that type 1 IgG subclasses (IgG3 >> IgG2b ≥ IgG2a) are superior in both opsonophagocytosis activity and complement activation than the type 2 (IgG1) subclass [44,46]. Nevertheless, some mouse IgG1 mAbs against bacterial CPSs have been reported to be opsonic and/or protective against *S. pneumoniae* and Group B *Streptococcus* [36,47]. How these mouse IgG1 can mediate protection is still the subject of ongoing research, and some new mechanisms are emerging, such as immunomodulatory functions [48] and bacterial gene expression alterations [49]. 

On the other hand, the two newly reported IgMs (mAbs 9E7 and 13C8) were shown to be opsonizing *in vitro*. However, in our passive immunization model, mAb 9E7 protects against challenge with a lethal dose of *S. suis* serotype 2, while mAb 13C8 confers no significant protection. This difference is also reflected in their respective OIs, but, surprisingly, does not correlate with their difference in avidity. Indeed, mAbs Z3 (low avidity) and 9E7 (strong avidity) are both highly opsonizing and protective while mAb 13C8 (intermediate avidity) is weakly opsonizing and non-protective in the conditions used. In the literature, correlation of protection with antibody avidity was reported for IgG, not for IgM [35,50,51,52]. In addition, relative avidities of the mAbs reported herein are weak compared to other published studies; which might also explain, at least in part, the lack of clear correlation between killing activity and avidity. Another explanation regarding the difference in behavior between mAbs 9E7/Z3 and mAb 13C8 may be their differing epitope preferences: the “poly-reactivity” of mAbs 9E7/Z3 might explain their ability to agglutinate *S. suis* serotype 2. As a matter of fact, agglutination by antibodies (notably IgM, but also IgG and IgA) greatly facilitates the removal of foreign pathogens and helps prevent the establishment of colonization and/or infection [33,53]. A study demonstrated that *S. pneumoniae* minimizes chain length to evade complement activation and gain advantage in a model of systemic infection, and how this effect can be overcome by antibody agglutination [54]. Other studies of pneumococcal agglutination using an IgM mAb have also suggested that complement is involved in bacterial immunoclearance by phagocytes and required for protection during *in vivo* challenges [34,55,56]. This bacterial clearance is indeed dependent of complement receptor 3 (CR3) as well as C3, while being independent of the Fcα/μ receptor [57]. Thus, it becomes clear that IgM-coated bacteria and agglutinated bacteria (with either IgM, IgG or IgA) can trigger complement-dependent phagocytosis. Overall, our results obtained with the three IgM mAbs suggest that opsonization by IgM and/or IgM-mediated bacterial agglutination might both help *S. suis* elimination by phagocytosis, which might explain the differences observed in their opsonic and/or avidity indexes. Data obtained by *in vitro* evaluation of opsonization and agglutination correlates with *in vivo* results and thus this approach might be valuable to predict protection *in vivo*. 

In the case of *S. suis*, while the precise epitopes to which the mAbs bind remain unknown, the differences in their functional characteristics *in vitro* and *in vivo* and their binding specifies to the different *S. suis* serotypes provide evidence that only certain CPS determinants might elicit protective antibodies; a crucial question to be answered in vaccine development. Since all mAbs were specific toward the sialylated CPSs, these findings demonstrate a dominance of the sialic acid-bearing side chain for the antibody response. This is in agreement with previous studies reporting that desialylated type 2 CPS is only weakly recognized by reference rabbit antiserum and that described serological cross-reactions between other sialylated serotypes (1, 1/2 and 14) would be mainly related by structural/conformational control by sialic acid [14,58]. The difference in specificity between mAbs 9E7 and 13C8 could possibly be an example of the influence played by the substitution of the β-Gal by a β-GalNAc in serotypes 1 and 1/2; whether this effect reflects the recognition of a structural or conformational epitope remains to be determined. Further studies will be required to allow exact determination of the epitopes recognized by these mAbs, and of the importance of each constituent of *S. suis* type 2 CPS. Nevertheless, our data indicates that antibodies targeting the sialic acid-bearing side chain ‘‘epitope’’ are protective against *S. suis* serotype 2.

In conclusion, using mice immunized with a glycoconjugate made from *S. suis* serotype 2 CPS, three new B cell hybridomas were isolated: Of these mAbs, two are IgMs and one is an IgG1 with different specificities against *S. suis* sialylated CPSs. Also, it was demonstrated that a glycoconjugate can induce protective antibodies against *S. suis* serotype 2 CPS that mediate *in vivo* bacterial killing by opsonophagocytosis. Thus, due to their described properties, these mAbs show potential for diagnostic and/or therapeutic purposes and provide fundamental knowledge of the CPS immunogenic determinants to further advance the development of optimized glycoconjugate vaccine against *S. suis*. By extension, the results presented herein provide new insights into the recognition of carbohydrate antigens by specific antibodies and how these properties can translate into protective immunity against encapsulated pathogens.

## 4. Materials and Methods

### 4.1. Bacterial Strains and Culture Conditions

*S. suis* strains that were used in this study are as follows: Serotype 2 strain S735; serotype 1 strain 1178027; serotype 1/2 strain 2651; serotype 14 strain DAN13730; and serotype 9 strain 1273590. All these strains were previously used for CPS structure determination [12,13,14,15]. They were included here as the target strains for *in vitro* opsonophagocytosis assays (OPA), an agglutination test, to prepare the heat-killed bacteria used in the dot-blot analyses and to purify the CPSs. *S. suis* serotype 2 strain P1/7 was used for the protection assay. Isolated colonies on sheep blood agar plates were inoculated in 5 mL of Todd–Hewitt broth (THB) (Becton Dickinson, Franklin Lakes, NJ, USA) and incubated for 8 h in a water bath at 37 °C with agitation at 120 rpm. Working cultures were prepared by transferring 10 µL of 8 h-cultures diluted 1:1000 with phosphate-buffered saline (PBS, pH 7.3) into 30 mL of THB, which was incubated for 16 h. Overnight cultures were washed twice with PBS and then resuspended in 30 mL of PBS (heat-killed bacteria and opsonophagocytosis assay) or THB (mouse protection assay). Bacterial solutions were appropriately diluted, and plated on Todd–Hewitt broth agar (THA, Becton Dickinson) to accurately determine bacterial concentrations using an Autoplate 4000 Spiral Plater (Spiral Biotech, Norwood, MA, USA). Heat-killed bacteria cultures were obtained as previously described [59]. Briefly, overnight cultures were washed twice with PBS and then resuspended in 30 mL of PBS. A sample was taken to perform bacterial counts on THA. Bacteria were immediately killed by incubating at 60 °C for 45 min and then were cooled on ice. Bacterial killing was confirmed by absence of growth on blood agar for 48 h.

### 4.2. Capsular Polysaccharide Purification and Mild Acid Hydrolysis

CPSs from *S. suis* serotypes 1, 1/2, 2, 14, and 9 were purified as previously described [12,13,14,15]. Except for serotype 9, desialylated CPSs were prepared by mild acid hydrolysis, and desialylation was confirmed by nuclear magnetic resonance, as previously described [12,13,14].

### 4.3. Monoclonal Antibody Production

All experiments involving mice were conducted in accordance with the guidelines and policies of the Canadian Council on Animal Care and the principles set forth in the Guide for the Care and Use of Laboratory Animals by the Animal Welfare Committee of the University of Montreal (protocol numbers rech-1399 and rech-1523). BALB/c mice (The Jackson Laboratory, Bar Harbor, ME) were immunized subcutaneously at day 0 with 25 µg of the CPS-TT glycoconjugate emulsified 1:1 (*v*/*v*) with TiterMax Gold^®^, as described [11]. Booster injections of 5 µg of the glycoconjugate emulsified with TiterMax Gold^®^ were given on days 21 and 35. Three days after the last booster immunization, mice were serologically tested with the indirect ELISA (positive reaction against serotype 2 CPS; see below). The spleen of the mouse showing the highest reaction (Total IgG plus IgM anti-CPS 2 titer of 4021) was recovered and fused with myeloma cell line SP2/0 using 50% (*w*/*v*) PEG 1500 (Sigma-Aldrich, Oakville, ON, Canada) as a fusogen [60]. Hybridoma supernatants were screened for the presence of anti-serotype 2 CPS antibodies using an indirect ELISA (see below). Positive hybridomas were cloned and then used to produce mAbs-containing supernatant. The immunoglobulin class of the mAbs in the culture supernatant was determined by anti-CPS ELISA titration using specific HRP-conjugates (see below).

### 4.4. Monoclonal Antibody Purification

Mouse IgM mAbs (Z3, 9E7, and 13C8) were purified by gel filtration chromatography using a XK16-100 column packed with Superdex 200 PG (GE Healthcare Life Sciences, Uppsala, Sweden), as described by Hale [61]. Firstly, culture supernatant containing mouse IgM was concentrated by 3 cycles of precipitation with ammonium sulfate, stirred and centrifuged. The precipitate was resuspended in PBS, injected unto the gel filtration column and eluted with 0.02 M NaHPO_4_ (pH 7.2), 0.15 M NaCl. The IgM mAbs (approx. 950 kDa) eluted in the fractions corresponding to the void volume. 

Mouse IgG1 mAb (16H11) was purified by affinity chromatography using Protein A Sepharose CL-4B resin (GE Healthcare Life Sciences). The column was equilibrated in binding buffer (0.5 M glycine/NaOH [pH 9.0], 3 M NaCl). Culture supernatant containing mouse IgG1 was mixed 1:1 with binding buffer, then applied to the column. Since protein A has a weak interaction with mouse IgG1, elution was carried in two steps. For the first step, elution was performed using 0.1 M sodium citrate (pH 5.5), where mouse IgG1 eluted, followed by a second step with 0.2 M glycine/HCl (pH 2.5) to remove all bound material. All eluted fractions were neutralized using 1 M Tris/HCl (pH 9.0).

Purified fractions containing anti-CPS mAbs (as identified by the indirect ELISA) were buffer-exchanged with PBS and concentrated using Amicon Ultra centrifugal filters (30K; EMD Millipore, Billerica, MA). Finally, purified mouse immunoglobulins were quantified by direct ELISA (see below).

### 4.5. Direct ELISA for Mouse Immunoglobulin (Ig) Quantification

To determine the amount of mouse Ig in either culture supernatants or purified fractions, samples were serially diluted with PBS and added to wells of an ELISA plate (Nunc-Immuno Polysorp; Canadawide Scientific, Toronto, ON, Canada). Standard curves ranging from 0–50 ng/mL were prepared in the same manner using either IgM or IgG1 isotype controls (Southern Biotech, Birmingham, AL, USA). After overnight coating at 4 °C, plates were washed with PBS containing 0.05% (*v*/*v*) Tween 20 (PBST) and blocked with PBS containing 1% (*w*/*v*) bovine serum albumin (BSA) (HyClone Laboratories, Logan, UT, USA) for 1 h. After washing, the plates were incubated for 1 h at room temperature with an HRP-conjugated isotype specific antibody (see below) diluted in PBST. The enzyme reaction was developed by addition of 3,3′,5,5′-tetramethylbenzidine (TMB; Invitrogen, Burlington, ON, Canada) and stopped after 30 min by addition of 0.5 M H_2_SO_4_, and the absorbance was read at 450 nm with an ELISA plate reader.

### 4.6. Indirect ELISA for Antibodies against Type 2 S. suis CPS

To perform the titration of anti-CPS mouse Ig isotypes and subclasses, 200 ng of native *S. suis* serotype 2 CPS in 0.1 M NaCO_3_ (pH 9.6) were added to wells of an ELISA plate. After overnight coating at 4 °C, plates were washed with PBST and blocked with PBS containing 1% (*w*/*v*) BSA for 1 h. After washing, culture supernatants or purified mAbs samples were serially diluted (2-fold) in PBST and were added to the wells and left for 1 h. After washing, antibodies were detected by incubating plates for 1 h with either HRP-conjugated goat anti-IgG plus IgM (H + L; Jackson Immunoresearch, West Grove, PA, USA), goat anti-IgG (Fcγ fragment specific; Jackson Immunoresearch), goat anti-IgM, goat anti-IgG1, goat anti-IgG2a, goat anti-IgG2b, or goat anti-IgG3 (Southern Biotech). The enzyme reaction was developed and read as described above. The reciprocal of the last dilution that resulted in an A_450_ equal to 0.1 (as a pre-established cutoff for comparison purposes) was considered to be the titer.

### 4.7. Avidity Measurement

The average avidity of mAbs was determined essentially as described previously [62]. Briefly, mAb dilutions producing absorbance readings at the top of the titration curve were incubated in duplicate wells coated with *S. suis* type 2 CPS (see above). Wells were then incubated for 15 min at room temperature with increasing concentrations (0 to 1 M) of the chaotrope, sodium thiocyanate (NaSCN; Sigma-Aldrich). Bound CPS is not released by treatment with NaSCN (data not shown). After incubation, NaSCN and eluted Ig were removed by extensive washings, and the wells were then incubated with HRP-conjugated goat anti-mouse IgM or IgG for 1 h (see above). The enzymatic reaction was developed until the A_450_ in the wells without NaSCN reached a value of 1.0. Since the avidity of the interaction is proportional to the resistance to elution by the chaotrope [63], avidities were expressed as avidity index (AI) (i.e., the millimolar concentration of NaSCN eluting 50% of CPS-specific mAbs). Calculations were performed as described by Goldblatt [64].

### 4.8. Dot-Blot

Dot-blot analyses were performed as described previously by Van Calsteren et al. [14]. Ten microliters of either heat-killed bacteria (at 1 × 10^9^ CFU/mL in PBS) or purified CPS (at 1 mg/mL in 50 mM NH_4_HCO_3_) for serotypes 1, 2, 1/2, 14, and 9 were blotted on a PVDF Western blot membrane (EMD Millipore). The membrane was blocked for 1 h with a solution of Tris-buffered saline (TBS) containing 2% casein, followed by 2 h of incubation with mAb culture supernatants whose titers ranged from 48 to 88, with an average (±SD) titer of 65.5 ± 16.9, as determined by anti-CPS indirect ELISA. The membrane was washed, and specific HRP-conjugated anti-mouse IgM or IgG (see above) was added for 1 h. The membrane was washed three times with TBS and revealed with a 4-chloro-1-naphthol solution (Sigma-Aldrich).

### 4.9. Opsonophagocytosis Assay (OPA)

Murine whole blood OPAs were performed as previously described [11,31], with some modifications. Blood was collected by intracardiac puncture from naive CD-1 mice (Charles River, Wilmington, MA), treated with sodium heparin (Sigma-Aldrich), and then diluted to obtain 6.25 × 10^6^ leukocytes/mL in RPMI 1640 supplemented with 5% heat-inactivated fetal bovine serum, 10 mM HEPES, 2 mM L-glutamine, and 50 mM 2-mercaptoethanol. All reagents were from Gibco (Invitrogen). All blood preparations were kept at room temperature. Using washed bacterial cultures grown as described above, final bacterial suspensions were prepared in complete cell culture medium to obtain a concentration of 1.25 × 10^6^ CFU/mL. The number of CFU/mL in the final suspension was determined by plating samples as described above. All bacterial preparations were kept on ice. 

To evaluate the opsonizing capacity of the mAbs, samples were prepared by diluting to the desired concentration (indicated in the figure legends) the purified mAbs or an IgM isotype control (clone MM-30; Biolegend, San Diego, CA, USA) in complete cell culture medium to 80 µL (40% *v/v*). To the diluted mAb samples were added 80 µL of whole blood (5 × 10^5^ leukocytes) and 40 µL of either the *S. suis* serotype 2, 1, 1/2, 14 or 9 strain (5 × 10^4^ CFU; multiplicity of infection [MOI] = 0.1) in a microtube to a final volume of 0.2 mL. A tube with complete cell culture medium (no antibody), whole blood and bacteria was similarly prepared as a negative control to determine the bacterial killing percentage for the mAbs. The tube tops were pierced using a sterile 25-gauge needle, and then the microtubes were incubated for 2 h at 37 °C with 5% CO_2_, with gentle manual agitation every 20 min. After incubation, viable bacterial counts were performed on THA using an Autoplate 4000 automated spiral plater. Also, tubes with addition of naive rabbit serum or serotype-specific rabbit anti-*S. suis* serum [65] were used as internal controls. The bacterial killing percentage was determined using the following formula: percentage of bacteria killed = [1 − (bacteria recovered from sample tubes/bacteria recovered from respective negative-control tubes)] × 100.

### 4.10. Bacterial Agglutination Assay

To evaluate the ability of the purified mAbs to agglutinate *S. suis* serotype 2, the OPA assay (see above) was adapted by replacing the 80 µL of whole blood with 80 µL of complete culture medium. The tubes contained 125 µg/mL of purified mAb and 5 × 10^4^ CFU in a final volume of 0.2 mL. A tube with complete cell culture medium (no antibody) and bacteria was similarly prepared as a negative control for agglutination. The tube tops were pierced using a sterile 25-gauge needle, and then the microtubes were incubated for 2 h at 37 °C with 5% CO_2_, with gentle manual agitation every 20 min. After incubation, viable bacterial counts were performed as described above. Also, tubes with addition of rabbit serum were used as internal controls (see above).

### 4.11. Phagocytosis Assay by Confocal Microscopy

The phagocytosis assay was adapted from [66]. J774A.1 murine macrophages (ATCC TIB-67; Rockville, MD, USA) were maintained in Dulbecco’s Modified Eagle’s Medium (Gibco) supplemented with 10% heat-inactivated fetal bovine serum (Gibco) and 100 U/mL penicillin/streptomycin (Gibco), and cells grown at 37 °C with 5% CO_2_. Confluent cell cultures were scraped, washed twice with PBS, suspended in antibiotic-free medium at 5 × 10^5^ cells/mL, and incubated for 3 h at 37 °C with 5% CO_2_ to allow cell adhesion onto 13 mm glass coverslips. Prior to the phagocytosis assay, to 80 µL of *S. suis* serotype 2 (5 × 10^5^ CFU; MOI = 1) suspension was added either 40 µL of control rabbit serum (see above) or purified mouse mAbs (final concentration of 125 µg/mL) and 80 µL (40%) of fresh naive mouse serum as a source of complement (final volume = 0.2 mL). Bacteria were opsonized for 30 min at 37 °C with end-over-end agitation. Then, pre-opsonized bacteria were added to macrophages and incubated for 1 h at 37 °C with 5% CO_2_ to allow optimal phagocytosis, determined during preliminary studies (data not shown). 

Coverslips were washed with PBS three times to remove non-associated bacteria. Cells were fixed and permeabilized with methanol/acetone (80:20) for 20 min at −20 °C. Cells were then washed and blocked for 10 min. Coverslips were incubated for 1 h with rabbit anti-*S. suis* serotype 2 serum. After washing, coverslips were incubated with Alexa-Fluor 647 (far-red) conjugated rat anti-mouse lysosomal-associated membrane protein-1 (LAMP-1) mAb (clone 1D4B, Biolegend) and with Alexa-Fluor 488 (green) conjugated goat anti-rabbit IgG (Invitrogen) for 1 h. Staining specificity was validated using appropriate controls (Figure S3). After washing, coverslips were incubated with DAPI for 5 min (blue; to stain the nuclei), washed and mounted on glass slides with mowiol containing DABCO. Samples were observed with an Olympus FluoView™ FV1000 confocal laser scanning microscope. Confocal microscopy images were obtained by scanning 3 times the image plane (x/y 63.121 µm; 0.079 µm/pixel), and analyzed using Fluoview software (Markham, ON, Canada).

### 4.12. Passive Immunization and Protection Assay of Mice

The protective capacity of the mAbs was tested in a standardized mouse model of infection using the virulent serotype 2 strain P1/7 as challenge strain [67,68]. The assay was repeated in two independent experiments (n = 10 each). Prior to the challenge, six-week-old male and female CD-1 mice (Charles River) were given a 0.2 mL intraperitoneal injection containing either the purified mAbs (at a dose of either 20 or 200 µg in PBS) or a type 2 specific rabbit antiserum prepared as described [65] (positive control; dilution 1/2 in PBS). Negative controls used were either PBS alone or an IgM isotype control (clone MM-30) at the highest dose (200 μg). One hour after passive immunization, mice were challenged with a LD90 dose of 1 × 10^8^ CFU of the virulent type 2 strain P1/7 by intraperitoneal inoculation. Mice were monitored at least three times daily until 72 h p.i., and at least twice daily thereafter until the end of the study (7 days p.i.) for clinical signs and mortality. Blood bacterial burden was assessed in surviving mice 24 h and 48 h p.i. by collecting 5 µL of blood from the caudal vein. Proper dilutions were plated as described above. 

### 4.13. Statistical Analyses

Parametric data are expressed as means ± standard errors of the means (SEM) and were analyzed for significance using one way analysis of variance (ANOVA) followed by the Tukey test. Non-parametric data are shown with the median and were analyzed for significance using the Kruskall-Wallis ANOVA on ranks followed by the Dunn procedure. Statistical analyses were performed using Systat SigmaPlot version 11.0, except for the survival curves analyses, which were performed using the log-rank (Mantel–Cox) test from GraphPad Prism version 6.01. A *p* < 0.05 was considered as statistically significant.

## Figures and Tables

**Table 1:** Summary of the properties of mouse mAbs directed against *S. suis* serotype 2 capsular polysaccharide.

	Z3	13C8	9E7	16H11
Isotype/Subclass	IgM	IgM	IgM	IgG1
Avidity index (mM)	24	74	127	157
Serotype specificity	2, 1, and 1/2	2 and 14	2, 1, 1/2, and 14	2
Opsonic index (µg/mL)	2.35	27.8	4.35	Non-opsonic
Agglutination	+	−	+	−
Phagocytosis	+	+	+	ND ^1^
Passive protection	+	+/−	+	−
Origin	[22]	This study	This study	This study

^1^ Not determined (ND).

## Supplementary Materials

The following are available online at https://www.mdpi.com/2076-0817/8/3/139/s1, Table S1: Anti-CPS titers of mAb-containing supernatants for the determination of their immunoglobulin classes. Figure S1: Specificity of mAbs for the capsular sialic acid. Figure S2: Passive immunization of CD-1 mice with murine IgG1 mAb 16H11 and survival after lethal challenge with *S. suis* type 2. Figure S3: Staining controls for the phagocytosis of *S. suis* serotype 2 by confocal laser scanning microscopy (Figure 7).
