# Supplementary material for: Characterization and Protective Activity of Monoclonal Antibodies Directed against Streptococcus suis Serotype 2 Capsular Polysaccharide Obtained Using a Glycoconjugate
Source: Pathogens. 2019 Sep 7;8(3):139. doi: 10.3390/pathogens8030139 (PMC6789524; doi:10.3390/pathogens8030139)
Supplement: Supplementary file 1 [file pathogens-08-00139-s001.pdf]

SUPPLEMENTARY MATERIAL

Characterization and protective activity of monoclonal antibodies directed against *Streptococcus suis* serotype 2 capsular polysaccharide obtained using a glycoconjugate

Guillaume Goyette-Desjardins [1,2,3,4], Sonia Lacouture [1,2], Jean-Philippe Auger [1,2,3], René Roy [2,3,4], Marcelo Gottschalk [1,2,3,4] and Mariela Segura [1,2,3,4]*

[1] Research Group on Infectious Diseases in Production Animals, Faculty of Veterinary Medicine, University of Montreal, St-Hyacinthe, Quebec, Canada
[2] Swine and Poultry Infectious Diseases Research Centre, Quebec, Canada
[3] Pharmaqam, Université du Québec à Montréal, Montreal, Quebec, Canada
[4] Canadian Glycomics Network (GlycoNet), University of Alberta, Edmonton, Alberta, Canada

* Correspondence: mariela.segura@umontreal.ca; Tel.: +1-450-773-8521 (ext. 0080)

Table S1. Anti-CPS titers of mAb-containing supernatants for the determination of their immunoglobulin classes.

**Antibody**<br>**Isotype / Subclass**	**Z3**	**13C8**	**9E7**	**16H11**
IgM	9084	107	112	0 [1]
IgG	0	0	0	89
IgG1	0	0	0	68
IgG2a	0	0	0	0
IgG2b	0	0	0	0
IgG3	0	0	0	0
**Conclusion**	IgM	IgM	IgM	IgG1

[1] 0 indicates that the titer is negative.

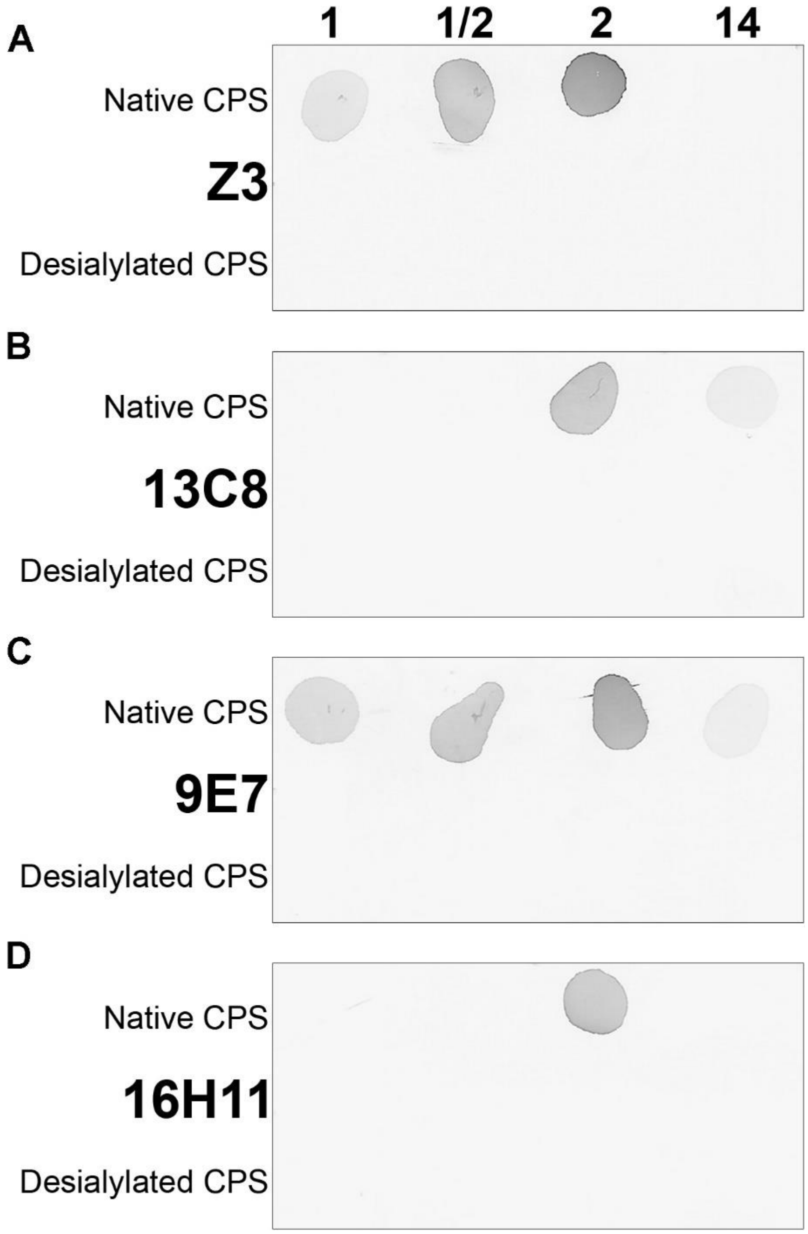

Figure S1. Specificity of mAbs for the capsular sialic acid. Dot-blot analyses with 10 µg of either native or desialylated CPSs from serotypes 1, 1/2, 2, or 14 using different mouse mAb culture supernatants: mAb Z3 (A), mAb 13C8 (B), mAb 9E7 (C), or mAb 16H11 (D).

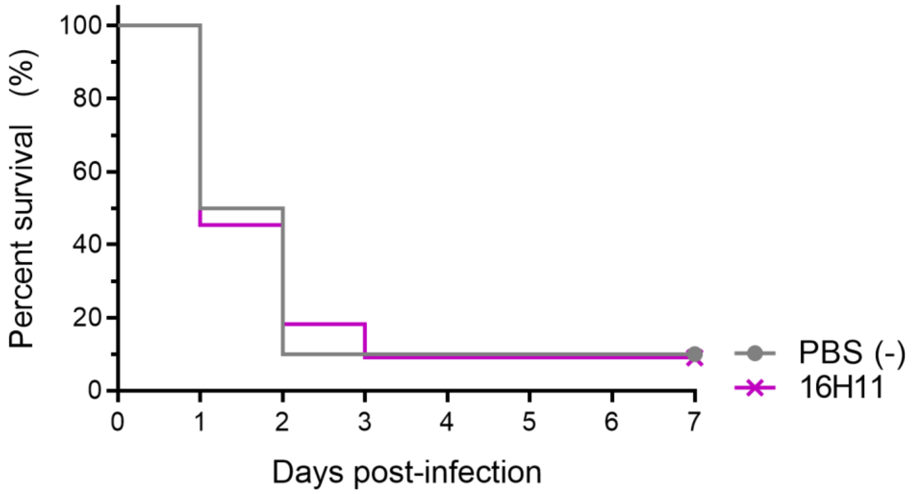

Figure S2. Passive immunization of CD-1 mice with murine IgG1 mAb 16H11 and survival after lethal challenge with *S. suis* type 2. Mice (n = 10) received doses of 20 µg of purified mAb intraperitoneally 1 h prior to the challenge. Mice were then challenged with a LD90 dose of 1 x 10^8 CFU of *S. suis* serotype 2 strain P1/7 by intraperitoneal inoculation. Negative control used for protection was PBS.

Figure S3. Staining controls for the phagocytosis of *S. suis* serotype 2 by confocal laser scanning microscopy (Fig. 7). *S. suis* opsonized with positive rabbit sera (A, B) or sterile culture media (C) was added to cells. After a bacteria–cell contact of 1 h, cells were fixed and stained as follows: (A) cells were labelled with rabbit serum against *S. suis* followed by an anti-rabbit IgG (Alexa-Fluor 488, green) then stained with DAPI (blue); (B) cells were labelled with serum against *S. suis* (no conjugate) and a mAb specific for LAMP-1 (Alexa-Fluor 647, red), then stained with DAPI; (C) cells were labelled with rabbit serum against *S. suis* followed by an anti-rabbit IgG (Alexa-Fluor 488) and a mAb specific for LAMP-1 (Alexa-Fluor 647), then stained with DAPI. Scale bar = 5 µm.

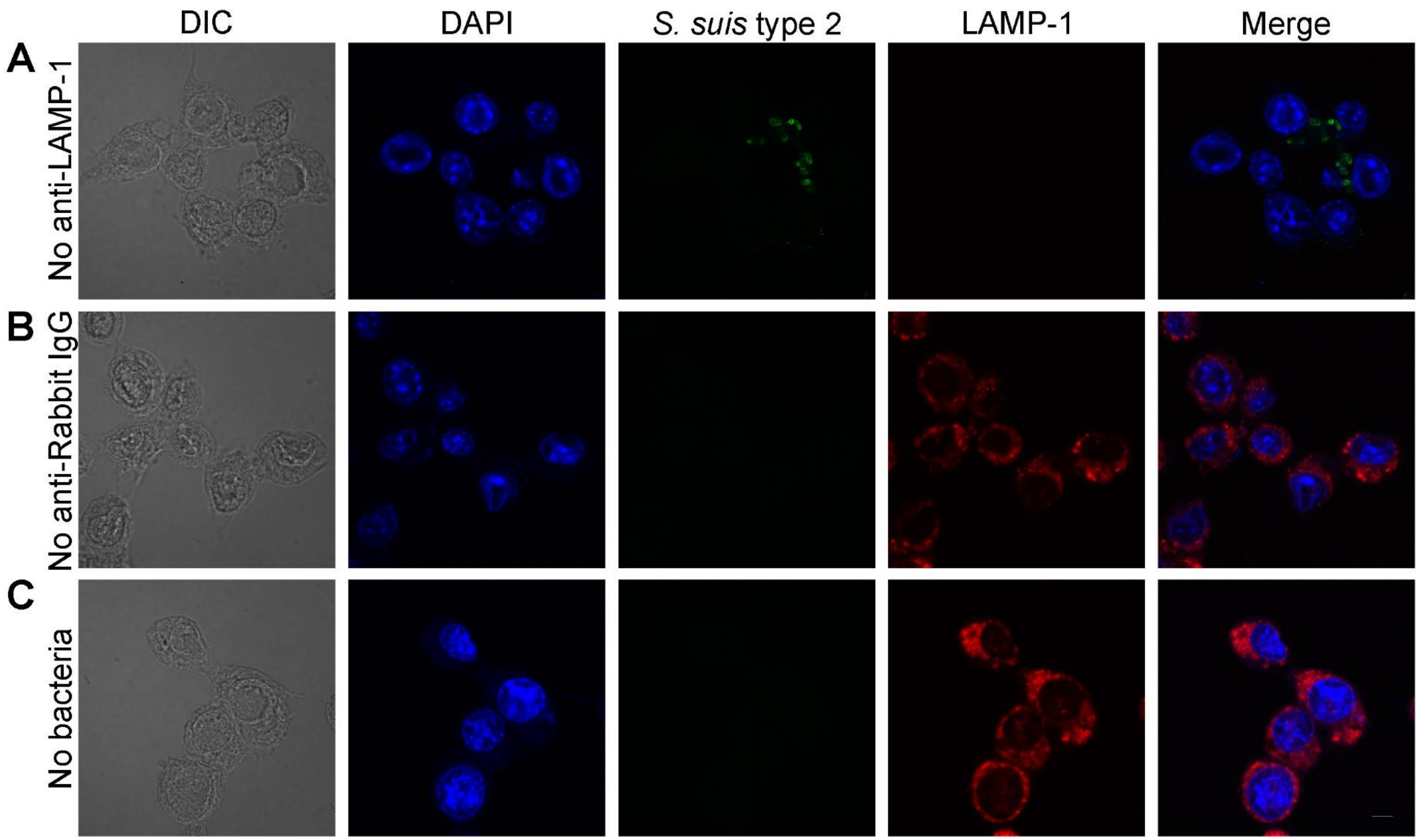
DIC
DAPI
S. suis type 2
LAMP-1
Merge
A
No anti-LAMP-1
B
No anti-Rabbit IgG
C
No bacteria

Figure S3
